# Genitourinary and Syphilitic Diseases

**Published:** 1904-03

**Authors:** Nelson W. Wilson

**Affiliations:** Buffalo, N.Y.; Genitourinary Surgeon to the Emergency Hospital


### PROGRESS IN MEDICAL SCIENCE.

### Genitourinary and Syphilitic Diseases.[1]

CONDUCTED BY NELSON W. WILSON, M. D., Buffalo, N.Y.
Genitourinary Surgeon to the Emergency Hospital.

### A YOUTHFUL GONORRHEIC.

GONORRHEA in the young is not so rare a disease as to excite comment or be worthy of more than passing mention because, as, in almost all cases, the method of infection can be traced to an extrasexual source. A careful search of all the available records, however, fails to reveal a case of gonorrhea, acquired in the usual way, in so young a person as the one herewith detailed, which was recently under the reporter's care at the Emergency Hospital. The patient, a boy aged nine, was poorly nourished and had the acutely developed cunning of a child of the streets. His home was in the lower section of the city both literally and figuratively, and his career has been the usual neglected life of such a child, whose parents gave him but spasmodic attention either moral, physical or mental. His appearance was that of the typical Italian child of nine years; his name was richly Irish and the apparent contradiction of sloe-black eyes and hair, Latinised features, and Gallic name was due to the fact that his mother was Portuguese and his father Irish.

This boy was received at the hospital in considerable pain because of his inability to urinate. His bladder was greatly distended and he said he had not urinated since the night before, it then being 11 o'clock in the morning. His penis was edematous, swollen, reddened and phymotic. The history in brief was that he had been under a physician's care the day before, that an effort had been made to catheterise him without success, and he had been recommended to the hospital for surgical treatment. An examination of his penis showed the presence of a large quantity of suspicious pus, which was stained and found to contain gonococci. His condition required immediate relief, hence, irrespective of the danger of carrying infection to the posterior urethra, the phimosis was cut and a catheter inserted into the bladder. A large quantity of cloudy urine was drawn and through the catheter he was given a bladder wash of permanganate of potash 1 to 8,000. His temperature was 102.4. He was put on urotropin .195 three times a day; he was catheterised every six hours and given a permanganate wash at the same time. His temperature rose within the next three days to the neighborhood of 105° and he could not voluntarily pass urine. On the fourth day he was put on uriseptin, a teaspoonful every three hours in hot water, and the urotropin was discontinued. The next day he urinated voluntarily but with some pain. His treatment now consisted of permanganate washes every six hours, increasing the strength gradually, and uriseptin internally, his bowels being kept open with salines, and he was put upon light diet.

From the time the uriseptin was given he began to improve, his urination became less and less painful and he made a rapid recovery, the gonococci disappearing during the sixth week after his admission. In the beginning he denied any sexual connection, but persistent questioning revealed the fact that he had enjoyed the society of a girl of twelve. This was so unusual that curiosity led to a visit of investigation, which disclosed the fact that the girl in question was gonorrheic and had been so for some time. The infection appeared to be prevalent in the neighborhood among the younger ones, and further investigation revealed its source to be a woman of public affection and of nymphomaniacal tendency.

This case is reported in narrative form because a detailed report would consume more space than is at command. The extreme youth of the patient and his acquired gonorrhea by the sexual route makes it interesting, as does the question which naturally arises as to whether he would have succeeded in his love affair at so early an age, had he not had in his make-up, the soft speech of the Irish, combined with the passion of the Portuguese.

### SUPRAPUBIC PROSTATECTOMY.

IN THE Lancet of recent date Sir William J. Collins reports a suprapubic prostatectomy in a man of seventy-one, who for two years had been suffering the torments of catheterisation. Under chloroform the bladder was irrigated and distended with warm boracic solution. Through a suprapubic incision a finger was introduced into the bladder and the membrane over the prostate stripped through. The gland was enucleated and, with two fingers in the rectum, was removed without further injury to the membrane. Hemorrhage which was not great was controlled with hot boracic solution. A drainage-tube was made fast in the suprapubic opening and a catheter inserted through the urethra. The latter was taken out on the second day because of irritation. In thirty-six days the suprapubic wound was healed and the patient discharged, cured of the condition for which he was operated.

This is the Freyer method and Collins advocates it. Why, is not apparent when one considers the excellent work done by American surgeons by the perineal route? Dr. Park's series of recent cases at the Buffalo General Hospital show an equally safe and satisfactory outcome. A late case was that of a man of seventy-six, who was received practically in a hopeless condition. He was suffering with uremic poison to a marked degree; his mind wandered for weeks prior to his admission; his urine had been dribbling and was extremely offensive. His case was complicated by the presence of multiple strictures of the urethra which had existed for many years.

Dr. Park did a perineal prostatectomy and removed a very much enlarged gland, fibrous in character; he also did internal urethrotomy after dividing the deep strictures externally, and left perineal drainage. The patient improved mentally for a few days and then returned to his former condition of flightiness. His urine showed no reason for the mental condition, and 33 centigramme doses of iodide of potash three times a day cleared him up in a few days and he was discharged cured, with the ability to control his urine. The suprapubic route is neither the route of common acceptance nor mechanics. In certain cases it is permissible and in very few others advisable. The concensus of opinion is largely in favor of perineal prostatectomy.

---

### ACUTE RHEUMATISM.

℞ Tongaline ........................................ ℥viii.

M. Sig.—A teaspoonful four or five times a day until pain is relieved. Then a teaspoonful three times a day.

